# 2,2′-Bis(4-nitro­phen­oxy)-1,1′-binaphth­yl

**DOI:** 10.1107/S1600536808016061

**Published:** 2008-06-07

**Authors:** Wen-Xian Liang, Zhi-Rong Qu

**Affiliations:** a Ordered Matter Science Research Center, College of Chemistry and Chemical Engineering, Southeast University, Nanjing 210096, People’s Republic of China

## Abstract

The title compound, C_32_H_20_N_2_O_6_, was synthesized by the reaction of 1,1′-binaphthyl-2,2′-diol and 4-nitro­phenol in the presence of K_2_CO_3_. The two naphthalene systems make a dihedral angle of 73.70 (5)°. The crystal packing involves mol­ecules connected by C—H⋯O hydrogen bonds into a chain along the *c* axis.

## Related literature

For the chemistry of 1,1′-binaphthyl-2,2′-diol, see: Hiroshi *et al.* (2005 ▶); Minatti & Dötz (2005 ▶); Pu (1998 ▶); Periasamy *et al.* (1998 ▶). 
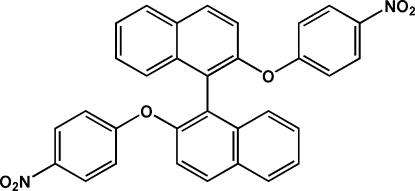


## Experimental

### 

#### Crystal data

C_32_H_20_N_2_O_6_
*M* *_r_* = 528.50
Monoclinic, 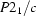

*a* = 7.6159 (9) Å
*b* = 24.810 (3) Å
*c* = 13.5022 (15) Å
β = 95.790 (3)°
*V* = 2538.3 (5) Å^3^
*Z* = 4
Mo *K*α radiation
μ = 0.10 mm^−1^
*T* = 296 (2) K
0.03 × 0.03 × 0.02 mm

#### Data collection

Bruker SMART APEX CCD area-detector diffractometer
Absorption correction: multi-scan (*SADABS*; Bruker, 2000 ▶) *T*_min_ = 0.988, *T*_max_ = 0.990
13574 measured reflections
4971 independent reflections
2521 reflections with *I* > 2σ(*I*)
*R*_int_ = 0.060

#### Refinement

*R*[*F* ^2^ > 2σ(*F* ^2^)] = 0.054
*wR*(*F* ^2^) = 0.114
*S* = 0.85
4971 reflections
361 parameters
H-atom parameters constrained
Δρ_max_ = 0.13 e Å^−3^
Δρ_min_ = −0.20 e Å^−3^

### 

Data collection: *SMART* (Bruker, 2000 ▶); cell refinement: *SAINT* (Bruker, 2000 ▶); data reduction: *SAINT*; program(s) used to solve structure: *SHELXS97* (Sheldrick, 2008 ▶); program(s) used to refine structure: *SHELXL97* (Sheldrick, 2008 ▶); molecular graphics: *SHELXTL* (Sheldrick, 2008 ▶); software used to prepare material for publication: *SHELXTL*.

## Supplementary Material

Crystal structure: contains datablocks I, global. DOI: 10.1107/S1600536808016061/kp2173sup1.cif

Structure factors: contains datablocks I. DOI: 10.1107/S1600536808016061/kp2173Isup2.hkl

Additional supplementary materials:  crystallographic information; 3D view; checkCIF report

## Figures and Tables

**Table 1:** Hydrogen-bond geometry (Å, °)

*D*—H⋯*A*	*D*—H	H⋯*A*	*D*⋯*A*	*D*—H⋯*A*
C16—H16*A*⋯O2^i^	0.93	2.54	3.425 (3)	159
C32—H32*A*⋯O6^ii^	0.93	2.45	3.360 (3)	165

Symmetry codes: (i) 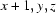; (ii) 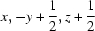.

**Fig. 1.**
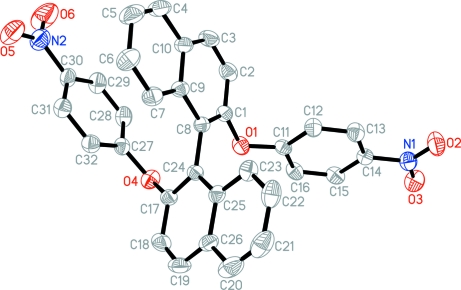
A view of the title compound with the atomic numbering scheme. Displacement ellipsoids were drawn at the 30% probability level. H atoms have been omitted for clarity.

## supplementary crystallographic information

### Comment

Axially chiral 2,2'-substituted-1,1'-binaphthyls have been extensively applied in many asymmetric processes, due to their highly stable chiral configuration with C_2_ symmetry (Pu, 1998). Chemists have synthesized a great number of 2,2'-substituted-1,1'-binaphthyls which have been used in asymmetric catalysis and chiral recognition (Periasamy *et al.*, 1998). Herein we report the structure of title compound, 2,2'-substituted-1,1'-binaphthyls (Fig. 1).

In the structure of the title compoud geometric parameters are in the usual ranges. The two naphthalene rings make a dihedral angle of 73.70 (5)°. The dihedral angles between the two 4-nitrobenzene rings and the two naphthalene rings directly attached to them (ring C11–C16 between ring C1–C10 76.42 (7)° and ring C27–C32 between ring C17–C26 81.99 (7)°, respectivley) are in synclinal range. The dihedral angles between the two 4-nitrobenzene rings and the two naphthalene rings, not directly attached to them, are remarkably different: between C27–C32 and C1–C10 is 22.62 (5)° and between C11–C16 and C17–C26 is 81.87 (6)°. The two aromatic rings with the smallest dihedral angle of 22.62 (5)°) exhibit weak intramolecular π–π interaction with the separation distance 3.694 (16) an 3.8227 (17) Å between the ring centroids of the 4-nitrobenzene rings and the two six-membered rings of the naphthalene ring. In the crystal structure no classical hydrogen bond is found. The crystal packing involves C—H···O hydrogen bonds generating chain along the *c* axis (Table 1, Fig. 2) and a weak C—H···π interaction [C20—H20···Cg (the ring C11, C12, C13, C14, C15, C16 with symmetry opeartion *x*, -*y* + 1,-*z* + 1) of 3.814 (3) Å].

### Experimental

1,1'-binaphthyl-2,2'-diol (1 mmol, 0.29 g) and 4-nitrophenol (2 mmol, 0.28 g) were dissolved in acetone (25 ml) in the presence of K_2_CO_3_ (1 mmol, 0.14 g) and refluxed for 2–3 days. After the mixture was cooled to room temperature, the solution was filtered and rotated in vacuum affording yellow precipitate of compound I. The crude product was recrystallized by slowly evaporating ethanol to yield colourless crystals.

### Refinement

All the H atoms were positioned geometrically and were allowed to ride on the C atoms to which they are bonded, with C—H = 0.93 Å, and with *U*_iso_(H) = 1.2*U*_eq_(C).

### Crystal data

**Table:**

C_32_H_20_N_2_O_6_	*F*(000) = 1096
*M**_r_* = 528.50	*D*_x_ = 1.383 Mg m^−^^3^
Monoclinic, *P*2_1_/*c*	Mo *K*α radiation, λ = 0.71073 Å
Hall symbol: -P 2ybc	Cell parameters from 1979 reflections
*a* = 7.6159 (9) Å	θ = 3.1–27.3°
*b* = 24.810 (3) Å	µ = 0.10 mm^−^^1^
*c* = 13.5022 (15) Å	*T* = 296 K
β = 95.790 (3)°	Block, colourless
*V* = 2538.3 (5) Å^3^	0.03 × 0.03 × 0.02 mm
*Z* = 4	

### Data collection

**Table:**

Bruker SMART APEX CCD area-detector diffractometer	4971 independent reflections
Radiation source: fine-focus sealed tube	2521 reflections with *I* > 2σ(*I*)
graphite	*R*_int_ = 0.060
φ and ω scans	θ_max_ = 26.0°, θ_min_ = 1.7°
Absorption correction: multi-scan (*SADABS*; Bruker, 2000)	*h* = −9→9
*T*_min_ = 0.988, *T*_max_ = 0.990	*k* = −23→30
13574 measured reflections	*l* = −16→14

### Refinement

**Table:**

Refinement on *F*^2^	Primary atom site location: structure-invariant direct methods
Least-squares matrix: full	Secondary atom site location: difference Fourier map
*R*[*F*^2^ > 2σ(*F*^2^)] = 0.054	Hydrogen site location: inferred from neighbouring sites
*wR*(*F*^2^) = 0.114	H-atom parameters constrained
*S* = 0.86	*w* = 1/[σ^2^(*F*_o_^2^) + (0.036*P*)^2^] where *P* = (*F*_o_^2^ + 2*F*_c_^2^)/3
4971 reflections	(Δ/σ)_max_ < 0.001
361 parameters	Δρ_max_ = 0.13 e Å^−^^3^
0 restraints	Δρ_min_ = −0.21 e Å^−^^3^

### Special details

**Table:**

Geometry. All e.s.d.'s (except the e.s.d. in the dihedral angle between two l.s. planes) are estimated using the full covariance matrix. The cell e.s.d.'s are taken into account individually in the estimation of e.s.d.'s in distances, angles and torsion angles; correlations between e.s.d.'s in cell parameters are only used when they are defined by crystal symmetry. An approximate (isotropic) treatment of cell e.s.d.'s is used for estimating e.s.d.'s involving l.s. planes.
Refinement. Refinement of *F*^2^ against ALL reflections. The weighted *R*-factor *wR* and goodness of fit *S* are based on *F*^2^, conventional *R*-factors *R* are based on *F*, with *F* set to zero for negative *F*^2^. The threshold expression of *F*^2^ > σ(*F*^2^) is used only for calculating *R*-factors(gt) *etc*. and is not relevant to the choice of reflections for refinement. *R*-factors based on *F*^2^ are statistically about twice as large as those based on *F*, and *R*- factors based on ALL data will be even larger.

### Fractional atomic coordinates and isotropic or equivalent isotropic displacement parameters (Å^2^)

**Table:**

	*x*	*y*	*z*	*U*_iso_*/*U*_eq_	
O4	1.3508 (2)	0.39839 (7)	0.26199 (13)	0.0562 (5)	
O1	0.9879 (2)	0.43838 (7)	0.17323 (12)	0.0553 (5)	
N1	0.4540 (3)	0.59046 (10)	0.10343 (16)	0.0583 (7)	
C25	1.0230 (4)	0.40498 (9)	0.44518 (18)	0.0446 (7)	
C8	1.0031 (3)	0.36017 (9)	0.27505 (17)	0.0397 (6)	
C24	1.1008 (3)	0.39169 (9)	0.35680 (17)	0.0399 (6)	
C11	0.8489 (4)	0.47360 (10)	0.15533 (17)	0.0448 (7)	
C17	1.2690 (4)	0.40833 (9)	0.34837 (19)	0.0448 (7)	
C23	0.8491 (4)	0.39068 (10)	0.46109 (19)	0.0529 (7)	
H23A	0.7797	0.3720	0.4118	0.063*	
C12	0.6795 (4)	0.46179 (10)	0.17407 (18)	0.0535 (7)	
H12A	0.6527	0.4279	0.1980	0.064*	
C7	1.0293 (4)	0.27455 (11)	0.37128 (19)	0.0553 (8)	
H7A	1.0782	0.2931	0.4273	0.066*	
O3	0.4934 (3)	0.63407 (8)	0.07056 (14)	0.0734 (6)	
C9	0.9767 (3)	0.30318 (10)	0.28363 (18)	0.0445 (7)	
C14	0.5925 (4)	0.54981 (11)	0.12233 (18)	0.0479 (7)	
C3	0.8464 (4)	0.30174 (12)	0.1116 (2)	0.0641 (8)	
H3A	0.7937	0.2828	0.0570	0.077*	
C1	0.9496 (3)	0.38393 (10)	0.18595 (19)	0.0467 (7)	
C27	1.3749 (3)	0.34527 (11)	0.23632 (19)	0.0462 (7)	
N2	1.4260 (4)	0.18672 (11)	0.1414 (2)	0.0768 (8)	
C15	0.7607 (4)	0.56203 (11)	0.10407 (18)	0.0558 (8)	
H15A	0.7872	0.5961	0.0808	0.067*	
C2	0.8700 (4)	0.35544 (12)	0.10426 (19)	0.0608 (8)	
H2A	0.8336	0.3733	0.0452	0.073*	
C22	0.7803 (4)	0.40373 (11)	0.5478 (2)	0.0671 (9)	
H22A	0.6655	0.3937	0.5571	0.081*	
C32	1.4198 (3)	0.30571 (11)	0.30582 (19)	0.0531 (7)	
H32A	1.4369	0.3143	0.3732	0.064*	
O2	0.3041 (3)	0.57870 (8)	0.12092 (16)	0.0825 (7)	
C10	0.9002 (4)	0.27407 (11)	0.2002 (2)	0.0526 (7)	
C16	0.8915 (4)	0.52370 (11)	0.12019 (17)	0.0540 (7)	
H16A	1.0066	0.5315	0.1076	0.065*	
C26	1.1244 (4)	0.43468 (10)	0.52074 (19)	0.0528 (7)	
C18	1.3671 (4)	0.43793 (10)	0.4217 (2)	0.0601 (8)	
H18A	1.4807	0.4491	0.4122	0.072*	
C13	0.5496 (4)	0.50002 (11)	0.15740 (19)	0.0578 (8)	
H13A	0.4343	0.4923	0.1696	0.069*	
C31	1.4392 (4)	0.25318 (11)	0.27459 (19)	0.0539 (7)	
H31A	1.4698	0.2259	0.3203	0.065*	
C19	1.2974 (4)	0.45038 (11)	0.5068 (2)	0.0663 (9)	
H19A	1.3642	0.4695	0.5564	0.080*	
C30	1.4125 (4)	0.24221 (11)	0.1749 (2)	0.0527 (7)	
C4	0.8843 (4)	0.21737 (12)	0.2078 (2)	0.0721 (9)	
H4A	0.8363	0.1978	0.1529	0.086*	
O5	1.4527 (3)	0.15122 (9)	0.20191 (16)	0.0954 (8)	
C20	1.0481 (5)	0.44692 (12)	0.6088 (2)	0.0767 (10)	
H20A	1.1141	0.4658	0.6591	0.092*	
C29	1.3761 (4)	0.28167 (12)	0.10560 (19)	0.0634 (8)	
H29A	1.3643	0.2733	0.0381	0.076*	
C28	1.3571 (3)	0.33380 (11)	0.13642 (19)	0.0564 (8)	
H28A	1.3323	0.3611	0.0900	0.068*	
C5	0.9374 (5)	0.19142 (12)	0.2932 (3)	0.0820 (10)	
H5A	0.9256	0.1542	0.2969	0.098*	
O6	1.4102 (4)	0.17823 (9)	0.05245 (17)	0.1275 (11)	
C6	1.0096 (4)	0.22003 (12)	0.3755 (2)	0.0680 (9)	
H6A	1.0451	0.2018	0.4344	0.082*	
C21	0.8817 (5)	0.43195 (13)	0.6221 (2)	0.0774 (11)	
H21A	0.8347	0.4405	0.6811	0.093*	

### Atomic displacement parameters (Å^2^)

**Table:**

	*U*^11^	*U*^22^	*U*^33^	*U*^12^	*U*^13^	*U*^23^
O4	0.0586 (14)	0.0454 (12)	0.0676 (12)	−0.0072 (9)	0.0207 (10)	0.0062 (10)
O1	0.0477 (13)	0.0515 (12)	0.0657 (12)	−0.0035 (10)	0.0005 (10)	0.0137 (10)
N1	0.0579 (19)	0.0617 (18)	0.0550 (15)	0.0040 (15)	0.0045 (14)	0.0064 (14)
C25	0.0507 (19)	0.0348 (15)	0.0473 (16)	0.0051 (13)	0.0006 (14)	0.0039 (13)
C8	0.0376 (17)	0.0417 (16)	0.0406 (15)	−0.0034 (12)	0.0087 (12)	−0.0034 (13)
C24	0.0422 (18)	0.0337 (15)	0.0429 (15)	−0.0030 (12)	−0.0007 (13)	0.0037 (12)
C11	0.0453 (19)	0.0494 (17)	0.0387 (15)	−0.0052 (14)	−0.0014 (13)	0.0036 (13)
C17	0.0462 (19)	0.0353 (15)	0.0519 (17)	−0.0057 (13)	−0.0001 (15)	0.0033 (13)
C23	0.055 (2)	0.0523 (18)	0.0518 (17)	0.0060 (14)	0.0094 (15)	0.0038 (14)
C12	0.050 (2)	0.0451 (18)	0.0666 (19)	−0.0043 (15)	0.0126 (16)	0.0111 (15)
C7	0.060 (2)	0.0493 (18)	0.0572 (18)	−0.0070 (15)	0.0095 (15)	0.0004 (15)
O3	0.0901 (18)	0.0586 (14)	0.0727 (13)	0.0070 (12)	0.0136 (12)	0.0199 (12)
C9	0.0418 (18)	0.0441 (17)	0.0490 (16)	−0.0071 (13)	0.0113 (13)	−0.0053 (14)
C14	0.049 (2)	0.0480 (18)	0.0461 (16)	−0.0017 (15)	0.0038 (14)	0.0031 (14)
C3	0.058 (2)	0.077 (2)	0.0542 (19)	−0.0067 (18)	−0.0057 (16)	−0.0206 (18)
C1	0.0399 (18)	0.0475 (18)	0.0529 (17)	−0.0023 (14)	0.0056 (14)	−0.0026 (15)
C27	0.0383 (17)	0.0463 (18)	0.0551 (17)	−0.0013 (13)	0.0094 (14)	0.0056 (15)
N2	0.093 (2)	0.067 (2)	0.0689 (19)	0.0145 (17)	0.0008 (17)	−0.0064 (17)
C15	0.057 (2)	0.0519 (18)	0.0573 (18)	−0.0111 (16)	0.0026 (16)	0.0150 (15)
C2	0.055 (2)	0.075 (2)	0.0502 (17)	0.0011 (17)	−0.0048 (15)	−0.0006 (17)
C22	0.071 (2)	0.069 (2)	0.065 (2)	0.0214 (18)	0.0218 (19)	0.0203 (18)
C32	0.056 (2)	0.0562 (19)	0.0463 (16)	−0.0008 (15)	0.0037 (14)	0.0003 (15)
O2	0.0545 (16)	0.0778 (16)	0.1164 (18)	0.0085 (12)	0.0148 (14)	0.0156 (13)
C10	0.0457 (19)	0.0504 (18)	0.0618 (18)	−0.0079 (14)	0.0058 (15)	−0.0147 (16)
C16	0.0465 (19)	0.0599 (19)	0.0551 (17)	−0.0085 (15)	0.0030 (14)	0.0144 (15)
C26	0.072 (2)	0.0379 (16)	0.0467 (17)	0.0052 (15)	−0.0054 (16)	−0.0045 (14)
C18	0.051 (2)	0.0488 (19)	0.079 (2)	−0.0140 (15)	−0.0049 (17)	0.0064 (17)
C13	0.049 (2)	0.0567 (19)	0.0702 (19)	−0.0057 (16)	0.0164 (16)	0.0074 (16)
C31	0.059 (2)	0.0557 (19)	0.0471 (17)	0.0056 (15)	0.0070 (14)	0.0049 (15)
C19	0.082 (3)	0.0444 (19)	0.068 (2)	−0.0130 (17)	−0.0175 (19)	−0.0091 (16)
C30	0.053 (2)	0.0541 (19)	0.0516 (17)	0.0122 (15)	0.0069 (15)	−0.0025 (16)
C4	0.073 (3)	0.053 (2)	0.089 (2)	−0.0150 (17)	0.007 (2)	−0.0250 (19)
O5	0.144 (2)	0.0593 (15)	0.0822 (16)	0.0152 (15)	0.0077 (15)	−0.0012 (13)
C20	0.117 (3)	0.061 (2)	0.050 (2)	0.012 (2)	0.002 (2)	−0.0108 (16)
C29	0.072 (2)	0.075 (2)	0.0436 (16)	0.0157 (18)	0.0068 (15)	0.0045 (17)
C28	0.059 (2)	0.061 (2)	0.0503 (18)	0.0101 (16)	0.0105 (15)	0.0154 (16)
C5	0.094 (3)	0.042 (2)	0.111 (3)	−0.0168 (19)	0.017 (2)	−0.007 (2)
O6	0.220 (3)	0.098 (2)	0.0606 (14)	0.0392 (19)	−0.0073 (17)	−0.0251 (14)
C6	0.086 (3)	0.048 (2)	0.072 (2)	−0.0035 (17)	0.0167 (19)	0.0086 (18)
C21	0.114 (3)	0.072 (2)	0.050 (2)	0.031 (2)	0.022 (2)	0.0027 (18)

### Geometric parameters (Å, °)

**Table:**

O4—C27	1.380 (3)	N2—O5	1.205 (3)
O4—C17	1.398 (3)	N2—O6	1.213 (3)
O1—C11	1.375 (3)	N2—C30	1.456 (3)
O1—C1	1.396 (3)	C15—C16	1.378 (3)
N1—O3	1.218 (2)	C15—H15A	0.9300
N1—O2	1.224 (3)	C2—H2A	0.9300
N1—C14	1.463 (3)	C22—C21	1.391 (4)
C25—C23	1.408 (3)	C22—H22A	0.9300
C25—C26	1.422 (3)	C32—C31	1.382 (3)
C25—C24	1.423 (3)	C32—H32A	0.9300
C8—C1	1.365 (3)	C10—C4	1.416 (3)
C8—C9	1.434 (3)	C16—H16A	0.9300
C8—C24	1.489 (3)	C26—C19	1.405 (4)
C24—C17	1.361 (3)	C26—C20	1.408 (3)
C11—C12	1.371 (3)	C18—C19	1.349 (3)
C11—C16	1.381 (3)	C18—H18A	0.9300
C17—C18	1.389 (3)	C13—H13A	0.9300
C23—C22	1.369 (3)	C31—C30	1.368 (3)
C23—H23A	0.9300	C31—H31A	0.9300
C12—C13	1.373 (3)	C19—H19A	0.9300
C12—H12A	0.9300	C30—C29	1.364 (3)
C7—C6	1.363 (3)	C4—C5	1.347 (4)
C7—C9	1.403 (3)	C4—H4A	0.9300
C7—H7A	0.9300	C20—C21	1.350 (4)
C9—C10	1.413 (3)	C20—H20A	0.9300
C14—C15	1.363 (3)	C29—C28	1.371 (3)
C14—C13	1.374 (3)	C29—H29A	0.9300
C3—C2	1.349 (3)	C28—H28A	0.9300
C3—C10	1.405 (3)	C5—C6	1.385 (4)
C3—H3A	0.9300	C5—H5A	0.9300
C1—C2	1.396 (3)	C6—H6A	0.9300
C27—C28	1.372 (3)	C21—H21A	0.9300
C27—C32	1.377 (3)		
			
C27—O4—C17	117.38 (19)	C1—C2—H2A	120.2
C11—O1—C1	118.0 (2)	C23—C22—C21	120.2 (3)
O3—N1—O2	123.4 (3)	C23—C22—H22A	119.9
O3—N1—C14	118.4 (3)	C21—C22—H22A	119.9
O2—N1—C14	118.2 (2)	C27—C32—C31	119.4 (2)
C23—C25—C26	118.2 (3)	C27—C32—H32A	120.3
C23—C25—C24	123.2 (2)	C31—C32—H32A	120.3
C26—C25—C24	118.7 (3)	C3—C10—C9	119.4 (2)
C1—C8—C9	117.7 (2)	C3—C10—C4	121.8 (3)
C1—C8—C24	120.6 (2)	C9—C10—C4	118.8 (3)
C9—C8—C24	121.4 (2)	C15—C16—C11	119.0 (3)
C17—C24—C25	118.3 (2)	C15—C16—H16A	120.5
C17—C24—C8	120.1 (2)	C11—C16—H16A	120.5
C25—C24—C8	121.6 (2)	C19—C26—C20	121.7 (3)
C12—C11—O1	123.6 (2)	C19—C26—C25	119.7 (3)
C12—C11—C16	120.9 (3)	C20—C26—C25	118.5 (3)
O1—C11—C16	115.4 (2)	C19—C18—C17	119.9 (3)
C24—C17—C18	123.0 (3)	C19—C18—H18A	120.1
C24—C17—O4	121.2 (2)	C17—C18—H18A	120.1
C18—C17—O4	115.8 (3)	C12—C13—C14	119.0 (3)
C22—C23—C25	121.2 (3)	C12—C13—H13A	120.5
C22—C23—H23A	119.4	C14—C13—H13A	120.5
C25—C23—H23A	119.4	C30—C31—C32	118.5 (3)
C11—C12—C13	119.9 (2)	C30—C31—H31A	120.7
C11—C12—H12A	120.1	C32—C31—H31A	120.7
C13—C12—H12A	120.1	C18—C19—C26	120.4 (3)
C6—C7—C9	121.0 (3)	C18—C19—H19A	119.8
C6—C7—H7A	119.5	C26—C19—H19A	119.8
C9—C7—H7A	119.5	C29—C30—C31	122.2 (3)
C7—C9—C10	118.2 (2)	C29—C30—N2	118.8 (3)
C7—C9—C8	122.4 (2)	C31—C30—N2	119.1 (3)
C10—C9—C8	119.3 (2)	C5—C4—C10	121.1 (3)
C15—C14—C13	121.5 (3)	C5—C4—H4A	119.5
C15—C14—N1	119.3 (3)	C10—C4—H4A	119.5
C13—C14—N1	119.2 (3)	C21—C20—C26	121.7 (3)
C2—C3—C10	121.0 (3)	C21—C20—H20A	119.1
C2—C3—H3A	119.5	C26—C20—H20A	119.1
C10—C3—H3A	119.5	C30—C29—C28	119.3 (3)
C8—C1—C2	123.0 (2)	C30—C29—H29A	120.3
C8—C1—O1	118.6 (2)	C28—C29—H29A	120.3
C2—C1—O1	118.2 (2)	C29—C28—C27	119.4 (3)
C28—C27—C32	121.0 (3)	C29—C28—H28A	120.3
C28—C27—O4	116.3 (2)	C27—C28—H28A	120.3
C32—C27—O4	122.7 (2)	C4—C5—C6	120.2 (3)
O5—N2—O6	122.5 (3)	C4—C5—H5A	119.9
O5—N2—C30	119.5 (3)	C6—C5—H5A	119.9
O6—N2—C30	118.0 (3)	C7—C6—C5	120.8 (3)
C14—C15—C16	119.7 (3)	C7—C6—H6A	119.6
C14—C15—H15A	120.2	C5—C6—H6A	119.6
C16—C15—H15A	120.2	C20—C21—C22	120.2 (3)
C3—C2—C1	119.5 (3)	C20—C21—H21A	119.9
C3—C2—H2A	120.2	C22—C21—H21A	119.9
			
C23—C25—C24—C17	178.9 (2)	C28—C27—C32—C31	−3.0 (4)
C26—C25—C24—C17	−0.3 (3)	O4—C27—C32—C31	179.0 (2)
C23—C25—C24—C8	−1.5 (4)	C2—C3—C10—C9	1.4 (4)
C26—C25—C24—C8	179.3 (2)	C2—C3—C10—C4	−176.9 (3)
C1—C8—C24—C17	−69.6 (3)	C7—C9—C10—C3	180.0 (2)
C9—C8—C24—C17	104.5 (3)	C8—C9—C10—C3	−2.0 (4)
C1—C8—C24—C25	110.8 (3)	C7—C9—C10—C4	−1.7 (4)
C9—C8—C24—C25	−75.1 (3)	C8—C9—C10—C4	176.3 (2)
C1—O1—C11—C12	17.3 (3)	C14—C15—C16—C11	−0.4 (4)
C1—O1—C11—C16	−165.2 (2)	C12—C11—C16—C15	0.0 (4)
C25—C24—C17—C18	−0.5 (4)	O1—C11—C16—C15	−177.5 (2)
C8—C24—C17—C18	179.9 (2)	C23—C25—C26—C19	−179.0 (2)
C25—C24—C17—O4	−177.6 (2)	C24—C25—C26—C19	0.2 (4)
C8—C24—C17—O4	2.8 (4)	C23—C25—C26—C20	1.9 (4)
C27—O4—C17—C24	−62.0 (3)	C24—C25—C26—C20	−178.9 (2)
C27—O4—C17—C18	120.7 (2)	C24—C17—C18—C19	1.4 (4)
C26—C25—C23—C22	−1.6 (4)	O4—C17—C18—C19	178.7 (2)
C24—C25—C23—C22	179.2 (2)	C11—C12—C13—C14	−0.2 (4)
O1—C11—C12—C13	177.6 (2)	C15—C14—C13—C12	−0.2 (4)
C16—C11—C12—C13	0.3 (4)	N1—C14—C13—C12	179.3 (2)
C6—C7—C9—C10	1.0 (4)	C27—C32—C31—C30	−0.2 (4)
C6—C7—C9—C8	−176.9 (2)	C17—C18—C19—C26	−1.5 (4)
C1—C8—C9—C7	179.0 (2)	C20—C26—C19—C18	179.8 (3)
C24—C8—C9—C7	4.7 (4)	C25—C26—C19—C18	0.7 (4)
C1—C8—C9—C10	1.1 (4)	C32—C31—C30—C29	3.3 (4)
C24—C8—C9—C10	−173.2 (2)	C32—C31—C30—N2	−177.7 (2)
O3—N1—C14—C15	0.9 (4)	O5—N2—C30—C29	−177.7 (3)
O2—N1—C14—C15	−179.6 (2)	O6—N2—C30—C29	2.5 (4)
O3—N1—C14—C13	−178.6 (2)	O5—N2—C30—C31	3.3 (4)
O2—N1—C14—C13	0.9 (4)	O6—N2—C30—C31	−176.5 (3)
C9—C8—C1—C2	0.5 (4)	C3—C10—C4—C5	179.6 (3)
C24—C8—C1—C2	174.8 (2)	C9—C10—C4—C5	1.3 (4)
C9—C8—C1—O1	−174.5 (2)	C19—C26—C20—C21	179.8 (3)
C24—C8—C1—O1	−0.2 (4)	C25—C26—C20—C21	−1.1 (4)
C11—O1—C1—C8	−117.7 (3)	C31—C30—C29—C28	−3.2 (4)
C11—O1—C1—C2	67.0 (3)	N2—C30—C29—C28	177.8 (3)
C17—O4—C27—C28	144.1 (2)	C30—C29—C28—C27	0.0 (4)
C17—O4—C27—C32	−37.9 (3)	C32—C27—C28—C29	3.1 (4)
C13—C14—C15—C16	0.5 (4)	O4—C27—C28—C29	−178.8 (2)
N1—C14—C15—C16	−179.0 (2)	C10—C4—C5—C6	−0.2 (5)
C10—C3—C2—C1	0.2 (4)	C9—C7—C6—C5	0.1 (4)
C8—C1—C2—C3	−1.2 (4)	C4—C5—C6—C7	−0.5 (5)
O1—C1—C2—C3	173.9 (2)	C26—C20—C21—C22	−0.1 (5)
C25—C23—C22—C21	0.5 (4)	C23—C22—C21—C20	0.4 (5)

### Hydrogen-bond geometry (Å, °)

**Table:**

*D*—H···*A*	*D*—H	H···*A*	*D*···*A*	*D*—H···*A*
C16—H16A···O2^i^	0.93	2.54	3.425 (3)	159.
C32—H32A···O6^ii^	0.93	2.45	3.360 (3)	165.

Symmetry codes: (i) *x*+1, *y*, *z*; (ii) *x*, −*y*+1/2, *z*+1/2.
